# Association Between Cross-Sectional Geometry and Cyclic Fatigue Resistance of Nickel–Titanium Endodontic Instruments: A Systematic Review

**DOI:** 10.3390/jfb17080399

**Published:** 2026-08-12

**Authors:** Mariya Kubatska, Julia Kensy, Joanna Cygankiewicz, Maja Gajewska, Anna Błaszczyk-Pośpiech, Kamil Wesołek, Agata Małyszek, Jacek Matys, Maciej Dobrzyński

**Affiliations:** 1 Kera Dental Clinic, Lilleakerveien 23b, 0283 Oslo, Norway; 2 Department of Pediatric Dentistry and Preclinical Dentistry, Wroclaw Medical University, Krakowska 26, 50-425 Wroclaw, Poland; julia.kensy@student.umw.edu.pl (J.K.); joanna.cygankiewicz@gmail.com (J.C.); m.gajewska97@gmail.com (M.G.); maciej.dobrzynski@umw.edu.pl (M.D.); 3 Pre-Clinical Research Center, Wrocław Medical University, Karola Marcinkowskiego 1, 50-368 Wroclaw, Poland; anna.blaszczyk-pospiech@umw.edu.pl; 4 Józef Struś Multi-Specialty Municipal Hospital with a Care and Treatment Facility, Independent Public Health Care Facility, Szwajcarska 3, 61-285 Poznan, Poland; wesolekwesolek@o2.pl; 5 Department of Biostructure and Animal Physiology, Wrocław University of Environmental and Life Sciences, Kożuchowska 1, 51-631 Wroclaw, Poland; agata.malyszek@upwr.edu.pl; 6 Dental Surgery Department, Wroclaw Medical University, Krakowska 26, 50-425 Wroclaw, Poland

**Keywords:** cross-sectional geometry, cyclic fatigue, endodontic instruments, nickel–titanium, systematic review

## Abstract

This systematic review aimed to evaluate whether cross-sectional geometry is associated with cyclic fatigue resistance of nickel–titanium (NiTi) endodontic instruments and to identify the geometric and materials-related features most frequently associated with improved fatigue performance. The protocol was prospectively registered on the Open Science Framework (OSF). PubMed, Scopus, Embase, Web of Science, and WorldCat were searched in April 2026 in accordance with PRISMA 2020. Eligible studies were comparative in vitro investigations that explicitly evaluated cross-sectional geometry or a related geometric parameter as a prespecified study factor and reported quantitative cyclic fatigue outcomes. Of 107 screened records, 66 full-text reports were assessed and 23 studies were included in the qualitative synthesis. Twenty-two studies had a medium risk of bias and one had a low risk of bias according to the Quality Assessment Tool for In Vitro Studies (QUIN). Cyclic fatigue resistance was most often reported as time to fracture or number of cycles to failure. Instruments with reduced metal mass, smaller core volume, lower cross-sectional area, and greater flexibility tended to demonstrate higher fatigue resistance in curved canals. S-shaped and double-S-shaped cross-sections were most consistently associated with favorable outcomes; however, this association is more plausibly related to reduced bending stiffness and canal-wall contact forces than to increased intrinsic material fatigue strength. Flat-side designs did not show a consistent advantage. The evidence was limited by heterogeneous testing protocols and residual confounding by alloy, heat treatment, taper, manufacturing, surface condition, and kinematics. No meta-analysis or quantitative dimensional correlation was feasible because testing conditions and detailed geometric parameters were inconsistently reported.

## 1. Introduction

Root canal treatment is a fundamental procedure in modern dentistry, allowing teeth affected by irreversible pulpitis or pulp necrosis to be retained in the oral cavity rather than extracted. Successful endodontic treatment depends on effective chemomechanical preparation, disinfection, and obturation of the root canal system. Proper canal preparation enables the removal of infected tissue, facilitates irrigation, and creates conditions for long-term tooth function [1]. The development of nickel–titanium (NiTi) endodontic instruments significantly changed root canal preparation. Compared with traditional stainless-steel hand files, NiTi instruments offer greater flexibility, improved ability to negotiate curved canals, and more efficient shaping of the canal system. Contemporary NiTi systems include rotary and reciprocating instruments, single-file and multi-file systems, and instruments with different tapers, tip sizes, alloy treatments, and cross-sectional designs [2,3,4].

Despite these advantages, fracture of NiTi instruments remains an important clinical concern [5,6,7]. Instrument separation may occur mainly as a result of cyclic fatigue or torsional overload. Cyclic fatigue develops when an instrument rotates or reciprocates in a curved canal and is repeatedly subjected to alternating tensile and compressive stresses. Torsional failure occurs when the tip or another part of the instrument binds within the canal while the shank continues to rotate. Both mechanisms are influenced by anatomical conditions, operating parameters, alloy properties, surface quality, chemical environment, and instrument design [1,3,4,8,9,10].

The mechanical performance of NiTi instruments is strongly affected by metallurgical characteristics. Conventional NiTi instruments are predominantly austenitic and relatively stiff, whereas thermomechanically treated instruments may contain a higher proportion of martensite or R-phase. These phases are generally associated with lower elastic modulus, greater flexibility, and improved ability to accommodate repeated bending in curved canals. Technologies such as M-Wire, controlled memory wire, Gold treatment, Blue treatment, R-phase treatment, and electrical discharge machining (EDM) have therefore been introduced to improve mechanical behavior and reduce fracture risk [8,11,12]. Surface quality is also important because machining grooves, microcracks, and surface irregularities can act as stress-concentration sites and promote crack initiation [9].

In addition to metallurgy, instrument geometry plays a major role in mechanical behavior. Cross-sectional shape, core diameter, metal mass distribution, number and orientation of cutting edges, flute design, pitch, helical angle, and taper may all influence flexibility, cutting efficiency, debris removal, torsional resistance, and cyclic fatigue resistance [7,11]. Cross-sectional geometry is particularly relevant because it determines how metal is distributed within the instrument core. Instruments with smaller core volume and lower bending stiffness may adapt more easily to canal curvature, thereby reducing the amplitude of repeated bending stresses. However, a smaller cross-sectional area should not be interpreted as a direct increase in intrinsic fatigue strength; under the same applied external load, smaller sections may experience higher nominal stresses. Therefore, the effect of cross-sectional geometry must be interpreted under the specific loading condition of curved canals, where bending stiffness, canal-wall contact, stress concentration, and crack initiation interact.

**Figure 1:**
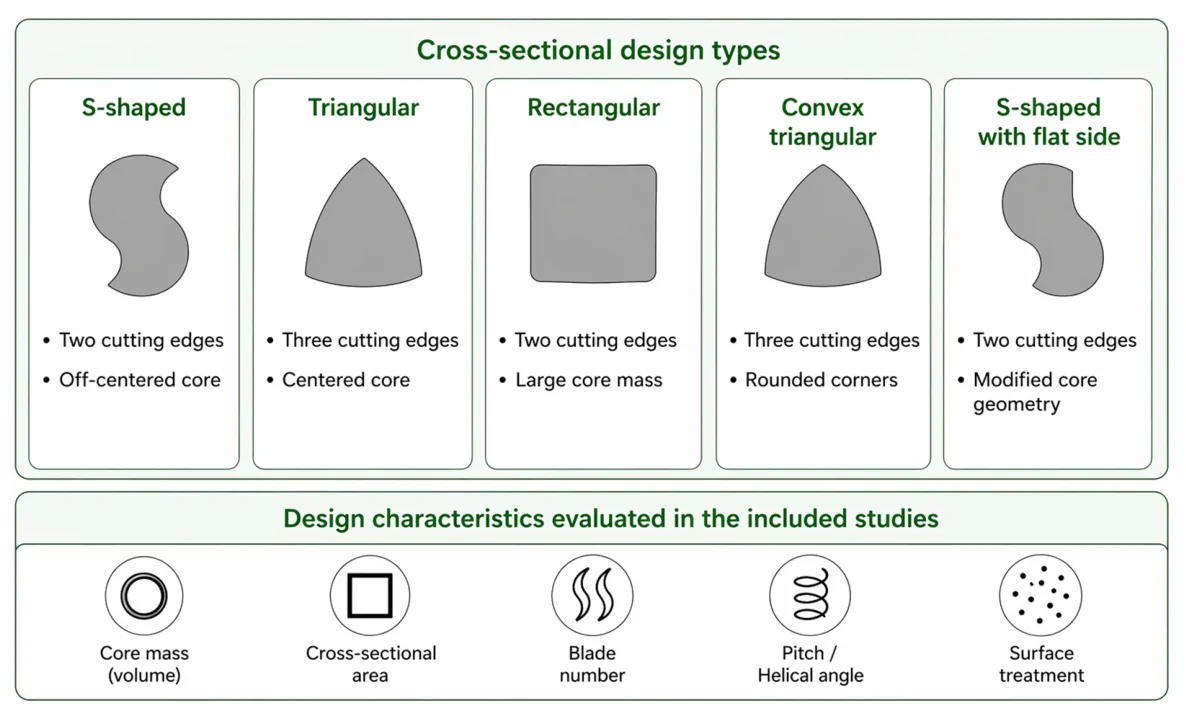
Cross-sectional designs of NiTi endodontic instruments and design characteristics evaluated in the included studies.

As summarized schematically in Figure 1, the included studies evaluated several cross-sectional configurations, including S-shaped, triangular, rectangular, convex triangular, and S-shaped designs with a flat side. Triangular and convex triangular designs should be distinguished because the latter presents rounded or convex borders and therefore a different distribution of core mass and stress concentration at the corners. These designs differ in the number of cutting edges, core position, core mass, corner configuration, and degree of lateral modification. However, the isolated effect of cross-sectional geometry is difficult to determine because commercially available systems usually differ simultaneously in alloy type, heat treatment, taper, size, manufacturing process, surface characteristics, and kinematics [2,3,4,5,13,14].

Therefore, although many studies have evaluated the mechanical properties and fracture mechanisms of NiTi instruments, the specific role of cross-sectional geometry in cyclic fatigue resistance remains difficult to interpret. The aim of this systematic review was to evaluate whether variation in cross-sectional geometry is associated with cyclic fatigue resistance of NiTi endodontic instruments and to identify which geometric features are most consistently associated with improved fatigue behavior. To preserve this focused scope, the synthesis was restricted to studies in which cross-sectional geometry or a related geometric parameter was an explicit study objective, prespecified comparison, or directly measured explanatory factor. The review also evaluates whether the observed influence of cross-sectional design can be interpreted independently or should be understood as an interaction between geometry, alloy phase composition, thermomechanical treatment, manufacturing technology, taper, surface condition, and motion kinematics [3,4,13,14,15,16,17].

## 2. Materials and Methods

### 2.1. Focused Question

This systematic review was conducted according to the PICO (Population, Intervention, Comparator, and Outcome) framework [18]. Similar structured frameworks have been used in previous dental systematic reviews to define the research question and organize the review methodology [19,20,21]. The focused question was: among NiTi endodontic instruments (P), is variation in cross-sectional geometry (I), compared with alternative cross-sectional designs (C), associated with differences in cyclic fatigue resistance (O)?

The primary outcome was cyclic fatigue resistance, reported as time to fracture (TTF), number of cycles to fracture/failure (NCF), or an equivalent quantitative fatigue-related parameter. Fracture and failure were considered equivalent when they referred to complete instrument separation during cyclic fatigue testing. NCF and TTF were interpreted together because NCF is derived from fracture time and instrument rotational speed or reciprocating cycle frequency. Direct comparison between studies was considered reliable only when test frequency, motion mode, canal curvature, temperature, and instrument size were comparable. Included studies were categorized as geometry-isolating studies or geometry-focused multifactorial studies according to whether major material and dimensional variables were controlled. Generic comparisons of commercial systems were not included solely because the systems had different known cross-sections. Because of heterogeneity in motion kinematics, test frequency, canal geometry, temperature, test models, and outcome reporting, the findings were synthesized narratively and were not statistically pooled.

### 2.2. Protocol

**Figure 2:**
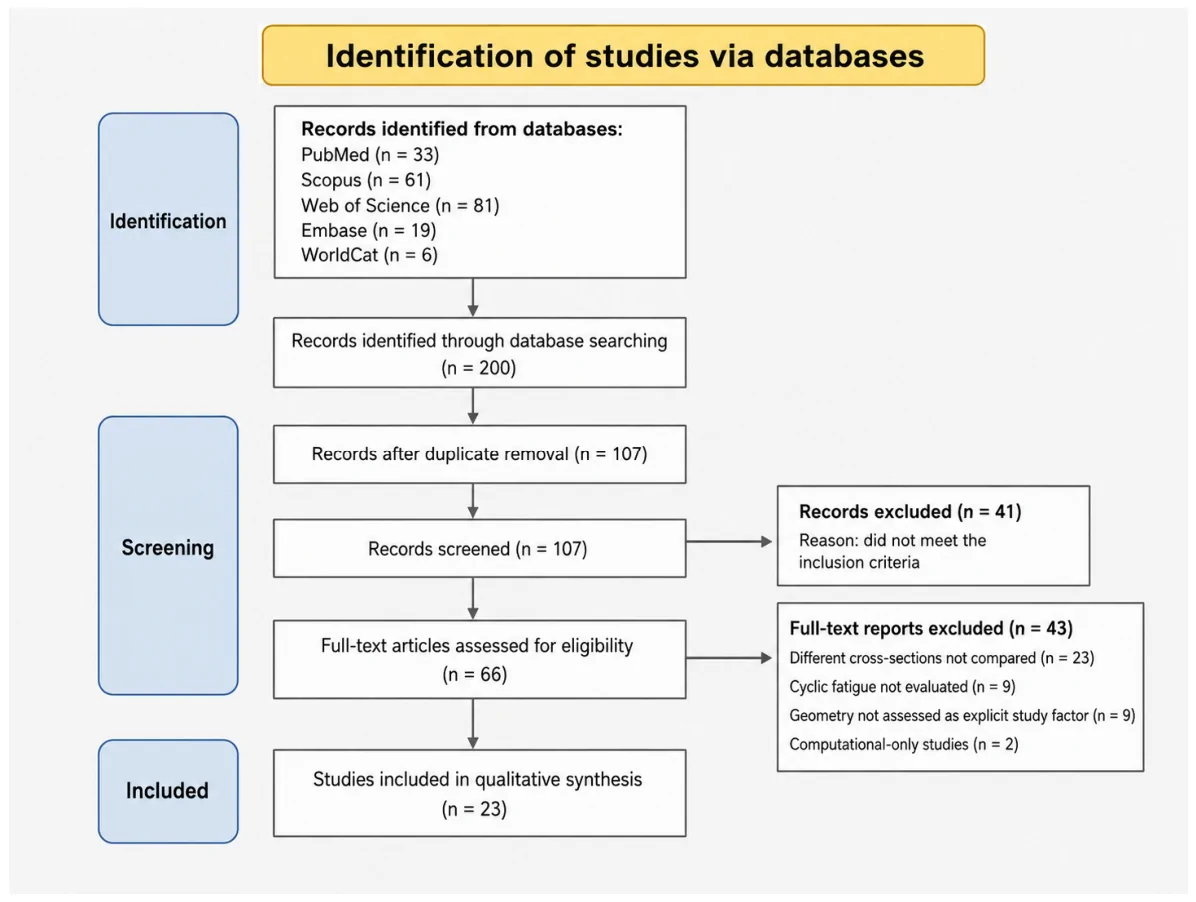
The PRISMA 2020 flow diagram [26].

The process of identifying and selecting studies adhered to the PRISMA methodology, as illustrated in Figure 2. Similar PRISMA-based methodological approaches have been applied in previous systematic reviews conducted by our research group [22,23,24,25]. The review protocol was prospectively registered on the Open Science Framework (OSF) at the following address: https://osf.io/3y2cm/overview (assessed on 10 May 2026). Reporting followed the PRISMA 2020 statement, and the completed checklist is provided as Supplementary Table S1.

### 2.3. Eligibility Criteria

The following criteria were employed to select the studies included in this review:

- Comparative studies of NiTi endodontic instruments in which cross-sectional geometry, cross-sectional area, core mass, core diameter, or a directly related geometric design feature was an explicit objective, prespecified comparison, or measured explanatory factor;
- Studies including at least two comparison groups with different cross-sectional geometries or directly quantified geometric characteristics;
- Studies reporting quantitative cyclic fatigue resistance as TTF, NCF, or an equivalent fatigue-related outcome;
- Experimental in vitro laboratory studies;
- Full-text studies published in English.

Exclusion criteria established by the reviewers included:

- Studies evaluating non-NiTi instruments, a single instrument without an eligible geometric comparator, or instruments with the same cross-sectional design when no other geometric parameter was directly assessed;
- Studies in which cross-sectional geometry was mentioned only as a post hoc explanation and was not an explicit study objective, prespecified comparison, directly measured variable, or analytical factor;
- Studies focused primarily on irrigant exposure, testing temperature, canal geometry, heat treatment, alloy type, kinematics, or general commercial-system performance without a planned analysis of cross-sectional geometry;
- Studies without quantitative cyclic fatigue testing; clinical or in vivo studies; computational-only studies without an experimental in vitro component; case reports; reviews; editorials; expert opinions; and commentaries;
- Duplicate publications or reports for which the full text was unavailable.

No publication year restrictions were applied. An original morphometric or imaging assessment of cross-sectional geometry was not required when the nominal cross-sectional design had been established in the study report, manufacturers’ specifications, or previous morphological publications. However, the mere presence of different known cross-sectional designs was not sufficient for inclusion when geometry was not an explicit objective, prespecified comparison, or analytical factor of the study. All potentially eligible articles were retrieved through institutional library access and available electronic resources. Where full texts could not be obtained despite these efforts, the studies would have been excluded during the study selection process and documented accordingly.

### 2.4. Information Sources, Search Strategy, and Study Selection

In April 2026, a comprehensive electronic search was conducted across multiple databases, including PubMed, Scopus, Web of Science (WoS), Embase, and WorldCat. The objective was to identify all pertinent in vitro studies examining the impact of the cross-sectional geometry of NiTi endodontic instruments on cyclic fatigue resistance. A combination of controlled vocabulary and free-text terms related to NiTi endodontic instruments, cross-sectional design, and cyclic fatigue was used. The search strategy and study selection workflow were designed in line with methodological procedures used in previous dental systematic reviews [27,28]. The general Boolean query applied was: (endodontic instruments OR endodontic files OR rotary files OR rotary instruments) AND (nickel-titanium OR NiTi OR nickel titanium) AND (cyclic fatigue OR cyclic fatigue resistance OR fatigue resistance) AND (cross-sectional design OR cross-sectional geometry OR cross section OR cross-sectional area OR instrument design).

Database-specific search strings were adapted as follows:

- PubMed: (endodontic instruments[Title/Abstract] OR endodontic files[Title/Abstract] OR rotary files[Title/Abstract] OR rotary instruments[Title/Abstract]) AND (nickel-titanium[Title/Abstract] OR NiTi[Title/Abstract] OR nickel titanium[Title/Abstract]) AND (cyclic fatigue[Title/Abstract] OR cyclic fatigue resistance[Title/Abstract] OR fatigue resistance[Title/Abstract]) AND (cross-sectional design[Title/Abstract] OR cross-sectional geometry[Title/Abstract] OR cross-section[Title/Abstract] OR cross-sectional area[Title/Abstract] OR instrument design[Title/Abstract])
- Scopus: TITLE-ABS-KEY (endodontic instruments OR endodontic files OR rotary files OR rotary instruments) AND (nickel-titanium OR NiTi OR nickel titanium) AND (cyclic fatigue OR cyclic fatigue resistance OR fatigue resistance) AND (cross-sectional design OR cross-sectional geometry OR cross-section OR cross-sectional area OR instrument design)
- Web of Science: TS = (endodontic instruments OR endodontic files OR rotary files OR rotary instruments) AND (nickel-titanium OR NiTi OR nickel titanium) AND (cyclic fatigue OR cyclic fatigue resistance OR fatigue resistance) AND (cross-sectional design OR cross-sectional geometry OR cross-section OR cross-sectional area OR instrument design)
- Embase: (endodontic instruments:ti,ab OR endodontic files:ti,ab OR rotary files:ti,ab OR rotary instruments:ti,ab) AND (nickel-titanium:ti,ab OR NiTi:ti,ab OR nickel titanium:ti,ab) AND (cyclic fatigue:ti,ab OR cyclic fatigue resistance:ti,ab OR fatigue resistance:ti,ab) AND (cross-sectional design:ti,ab OR cross-sectional geometry:ti,ab OR cross-section:ti,ab OR cross-sectional area:ti,ab OR instrument design:ti,ab)
- WorldCat: endodontic AND (NiTi OR nickel titanium) AND cyclic fatigue AND (cross-sectional design OR cross-sectional area)

The selection process followed the predefined eligibility criteria. Potentially eligible full texts were retrieved through institutional access and available electronic resources; full-text availability was treated as part of study retrieval rather than as an eligibility criterion.

### 2.5. Data Collection Process and Data Items

A team of six reviewers (J.K., K.W., A.B.-P., J.C., M.G., and M.K.) participated in screening, full-text eligibility assessment, and data extraction in accordance with the predefined criteria. All retrieved records were imported into Rayyan (Rayyan Systems Inc., Cambridge, MA, USA), where duplicates were identified using the software’s duplicate-detection function and verified manually before removal. For each eligible study, extracted information comprised the primary author, year, study design, cyclic fatigue outcome, instrument system, cross-sectional geometry or measured geometric parameter, alloy composition and/or heat treatment, instrument size and taper, manufacturing process and surface characteristics when reported, motion kinematics, test conditions, sample size, and additional mechanical outcomes. During the narrative synthesis, each comparison was evaluated in relation to these co-varying material, dimensional, manufacturing, surface, kinematic, and testing factors. Comparisons were classified as geometry-isolating only when the principal material and dimensional variables were matched or experimentally controlled; otherwise, they were interpreted as geometry-focused multifactorial comparisons. No statistical adjustment for these variables was attempted because the available study-level data and experimental protocols were insufficiently homogeneous. The information was documented in a standardized Microsoft Excel 365 spreadsheet (Version 2505, Build 16.0.18827.20102, 64-bit).

### 2.6. Risk of Bias and Quality Assessment

During title/abstract screening and full-text assessment, each record was independently evaluated by two reviewers selected from the six-member team (M.K., J.K., J.C., M.G., A.B.-P., and K.W.). Disagreements were resolved through discussion, and a third reviewer was consulted when consensus could not be reached. During the revision, the eligibility criteria were operationally clarified to require cross-sectional geometry or a directly related geometric parameter to be an explicit study factor; the focused question and database search strategy were not changed.

The methodological quality and risk of bias of each included study were independently evaluated by two reviewers (J.M. and M.D.) using the Quality Assessment Tool for In Vitro Studies (QUIN) [29]. The QUIN tool was selected because it was specifically developed and validated for assessing methodological quality and risk of bias in in vitro dental studies. In the present review, it was used as a structured appraisal of methodological features and reporting quality and not as a formal measure of the certainty of evidence. The QUIN tool comprises 12 criteria evaluating key methodological domains. Each criterion was scored as 2 (“adequately specified”), 1 (“inadequately specified”), or 0 (“not specified”). For each study, the final QUIN score was calculated using the following formula:

Final score (%) = (Total score obtained × 100)*/*(2 × number of applicable criteria).

Based on the final percentage score, studies were classified as having a low risk of bias (>70%), medium risk of bias (50–70%), or high risk of bias (<50%).

To evaluate consistency between raters, Cohen’s kappa was calculated using MedCalc (version 23.1.7, MedCalc Software Ltd., Ostend, Belgium). The kappa coefficient was 0.7 (*p* < 0.001), indicating substantial agreement. Reporting bias and certainty of evidence were not formally assessed because no meta-analysis was performed and the small, methodologically heterogeneous body of in vitro evidence did not permit a reliable outcome-level assessment.

## 3. Results

### 3.1. Study Selection

After duplicate removal, 107 records were screened; 41 were excluded during title and abstract screening, and 66 full-text reports were assessed. Forty-three full-text reports were excluded: 23 did not compare instruments with different cross-sectional geometries, nine did not evaluate cyclic fatigue resistance, nine reported cyclic fatigue but did not assess cross-sectional geometry or a directly related geometric parameter as an explicit study factor, and two were computational-only studies without an experimental in vitro component. Consequently, 23 studies were included in the qualitative synthesis. Study-level characteristics of the 23 included studies are presented in Supplementary Table S2.

### 3.2. General Characteristics of the Included Studies

All 23 included studies were comparative in vitro laboratory investigations that treated cross-sectional geometry or a directly related geometric parameter as an explicit study factor and reported quantitative cyclic fatigue outcomes [30,31,32,33,34,35,36,37,38,39,40,41,42,43,44,45,46,47,48,49,50,51,52]. Cyclic fatigue was evaluated primarily in artificial canals with standardized curvature angles and radii, commonly ranging from 30° to 90° and from 3 to 10 mm, respectively. Some studies additionally used extracted teeth, simulated resin canals, or micro-CT-based shaping assessment [31,37,50,51]. The most common outcomes were TTF and NCF. Because these measures depend on rotational speed or reciprocating frequency and were obtained under non-equivalent experimental conditions, pooled estimates and I^2^ statistics were not calculated.

In addition to cyclic fatigue testing, included studies evaluated torsional resistance, maximum torque, angular deflection, bending resistance or flexibility, buckling resistance, microhardness, cutting efficiency, or shaping performance [30,31,32,33,34,35,36,37,38,39,40,41,42,43,44,45,46,47,48,49,50,51,52]. Fracture surfaces were commonly examined by scanning electron microscopy to confirm cyclic or torsional fracture features [30,31,32,33,35,37,38,41,42,43,44,45,46,48,49,50,51]. Several studies also incorporated metallurgical or structural analyses, including differential scanning calorimetry, energy-dispersive X-ray spectroscopy, X-ray diffraction, micro-computed tomography, optical or confocal microscopy, nanoindentation, or surface evaluation [32,33,34,35,36,38,39,40,41,42,43,44,45,46,47,48,49,50,51,52].

The included evidence covered rotary, reciprocating, single-file, multi-file, and glide-path instruments with a wide range of cross-sectional designs, alloy treatments, sizes, and tapers [30,31,32,33,34,35,36,37,38,39,40,41,42,43,44,45,46,47,48,49,50,51,52]. Most studies evaluated instruments with an ISO tip size of 25 and tapers between 0.04 and 0.08, although larger instruments, variable-taper systems, and glide-path files were also represented. Methodologically, the included evidence comprised geometry-isolating studies and geometry-focused multifactorial studies. Geometry-isolating studies compared instruments with similar dimensions and metallurgical treatment while varying the cross-section or directly quantified cross-sectional area, core mass, or lateral flattening. Geometry-focused multifactorial studies explicitly incorporated geometric design into their objectives or analyses but also evaluated alloy, heat treatment, taper, manufacturing, or kinematics. Generic commercial-system comparisons were not retained solely because the tested instruments had different known cross-sections.

The study-level summary has been moved to Supplementary Table S2 and organized alphabetically by the first author’s surname.

### 3.3. Main Study Outcomes

#### 3.3.1. Overall Effect of Cross-Sectional Geometry

The synthesis indicates that cross-sectional geometry is associated with cyclic fatigue resistance primarily through metal mass distribution, core size, cross-sectional area, bending stiffness, and flexibility [32,35,38,41,43,44,47]. Instruments with reduced metal mass and smaller cross-sectional area generally showed higher cyclic fatigue resistance than more massive and rigid designs [32,41,44,47]. This trend was strongest in studies with similar instrument dimensions or direct assessment of cross-sectional area, core mass, or metal volume [32,41,44,47].

Cyclic fatigue differences were therefore interpreted according to study context. When commercial systems were compared, the observed outcomes were considered multifactorial and not attributed to cross-sectional geometry alone.

However, this relationship should not be interpreted as a simple area–strength relationship. Under the same externally applied load, a smaller cross-sectional area may increase nominal stress. In curved canals, the more relevant mechanism is that a reduced core mass lowers bending stiffness, decreases restoring forces against the canal wall, and may reduce the amplitude of alternating tensile-compressive stresses during rotation or reciprocation. Therefore, the beneficial effect of a smaller or less massive cross-section is most plausible under curvature-controlled bending conditions, rather than as a direct increase in intrinsic material fatigue strength.

The available evidence suggests that the amount and distribution of metal within the instrument core are more informative than nominal cross-sectional shape alone [32,41,44,47]. Reduced core mass was generally associated with greater flexibility and improved resistance to repeated bending in curved canals [32,41,44,47]. Larger core dimensions and greater cross-sectional area increased stiffness and torsional strength but were frequently associated with lower cyclic fatigue resistance [32,44]. Thus, the most consistent finding is not that one nominal shape is universally superior, but that designs reducing metal mass while preserving torsional safety tend to perform better under cyclic fatigue loading [32,35,41,44,47].

#### 3.3.2. S-Shaped and Double-S-Shaped Designs

S-shaped and double-S-shaped cross-sections were most frequently associated with favorable cyclic fatigue performance [31,32,35,38,41,43,44,45,47]. This pattern occurred across rotary, reciprocating, and glide-path instruments and was commonly accompanied by lower core mass, reduced cross-sectional area, or greater flexibility [31,32,35,41,43,44,47]. 

Instruments with these designs often showed longer TTF or higher NCF than instruments with more massive triangular, convex triangular, rectangular, or square configurations [31,32,35,38,41,43,44,45,47]. Improved angular deflection or flexibility provided a plausible mechanical explanation [32,41,43,44].

However, superiority was not universal. Triangular, rectangular, trapezoidal, or variable designs also achieved favorable fatigue behavior when combined with appropriate alloy treatment, taper, manufacturing, or optimized core geometry [33,36,37,39,50,51]. S-shaped geometry should therefore be interpreted as a favorable design pattern, not an independent guarantee of superior resistance.

#### 3.3.3. Flat-Side, Laterally Flattened, Triangular, and Rectangular Designs

Flat-side or laterally flattened designs did not show a consistent cyclic fatigue advantage [30,31,52]. In the eligible studies, these instruments did not consistently outperform conventional or S-shaped designs and sometimes showed lower fatigue resistance [30,31,52]. Lateral flattening alone therefore cannot be regarded as a reliable strategy for improving cyclic fatigue behavior.

Triangular, convex triangular, rectangular, parallelogram, square, and variable cross-sections showed more heterogeneous outcomes [32,33,34,36,37,39,44,46,50,51]. Favorable results for non-S-shaped designs were generally observed when geometry interacted with controlled-memory alloys, heat treatment, EDM manufacturing, optimized taper, or reduced core mass [33,36,37,39,50,51]. Their performance should consequently be interpreted through the combined effects of geometry, alloy, taper, manufacturing process, and kinematics.

#### 3.3.4. Trade-Off Between Cyclic Fatigue Resistance and Torsional Resistance

A recurring finding was the trade-off between cyclic fatigue and torsional resistance [32,44,50,51]. Instruments with smaller cross-sectional area and lower metal mass tended to be more flexible and more resistant to cyclic fatigue, whereas larger sections tended to show greater torque resistance and stiffness [32,47]. This balance is clinically relevant because design features that improve performance in curved canals may reduce resistance to torsional overload in narrow or constricted canals. Cross-sectional geometry should therefore be interpreted in relation to the intended mechanical balance of the instrument rather than as an isolated ranking characteristic.

#### 3.3.5. Influence of Alloy, Heat Treatment, and Manufacturing Technology

The included evidence showed that geometry alone did not fully explain cyclic fatigue resistance [33,36,37,39,50,51,52]. Alloy phase composition, transformation temperatures, thermomechanical treatment, surface condition, and manufacturing technology modified the behavior of instruments with similar or different cross-sectional designs [33,36,37,39,50,51,52]. Conventional austenitic NiTi instruments are generally stiffer, whereas instruments containing martensite or R-phase usually exhibit greater flexibility and improved accommodation of repeated bending.

Instruments combining flexible alloys with variable or optimized cross-sectional designs frequently showed high cyclic fatigue resistance even when their nominal cross-section was rectangular, parallelogram-shaped, square, or triangular [33,36,37,39,50,51]. A favorable cross-sectional design may improve resistance, but its effect can be strengthened, weakened, or outweighed by alloy treatment, taper, and manufacturing technology.

#### 3.3.6. Influence of Motion Type and Repeated Use

Motion type also modified cyclic fatigue resistance and could influence the apparent effect of cross-sectional geometry [41,43,44,52]. Comparisons involving rotary and reciprocating instruments were interpreted cautiously because observed differences may reflect both geometry and kinematics. The available evidence regarding repeated use was limited and inconsistent for broad clinical recommendations [40]. Repeated use was associated with a reduction in cyclic fatigue resistance in the study that directly assessed this factor, although the magnitude of this effect varied between systems [40]. Therefore, no universal conclusion can be drawn regarding the safe number of uses based only on the included evidence. The evidence-based interpretation of cyclic fatigue resistance across the included studies is summarized in Table 1 and Supplementary Table S2.

### 3.4. Quality Assessment

Based on the QUIN assessment of the 23 included studies, 22 were classified as having a medium risk of bias: nine scored 58.33% [34,35,39,41,42,45,46,47,48] and 13 scored 66.67% [30,32,33,36,37,38,40,43,44,49,50,51,52]. One study [31] scored 75.00% and was classified as having a low risk of bias. No included study was classified as having a high risk of bias (Table 2).

## 4. Discussion

The aim of this systematic review was to determine whether cross-sectional geometry is associated with cyclic fatigue resistance of NiTi endodontic instruments and to identify which design features are most frequently associated with improved mechanical performance. Restricting the synthesis to studies that explicitly evaluated geometry or a directly related geometric parameter strengthened alignment between the focused question, eligibility criteria, and included evidence. Higher cyclic fatigue resistance was most consistently associated with reduced metal mass, lower core volume, smaller cross-sectional area, and greater flexibility, features commonly observed in S-shaped or double-S-shaped designs [31,32,35,38,41,43,44,45,47]. Geometry nevertheless did not act independently: several eligible studies also demonstrated important interactions with alloy, heat treatment, taper, manufacturing, surface condition, and kinematics [33,36,37,39,50,51,52]. The principal contribution of this review is therefore an interpretative framework for distinguishing findings that can reasonably be associated with geometry from findings that remain inseparable from co-varying material, dimensional, manufacturing, and operating factors. Publications that did not satisfy the predefined eligibility criteria were not included in the systematic synthesis. This included studies without cyclic fatigue testing, studies of a single system or the same cross-section, computational-only studies, and generic commercial-system comparisons in which geometry was not an explicit study factor. Where such publications are cited in the Discussion [36,45,48,53,54,55,56,57,58,59,60,61,62,63,64,65,66,67,68,69,70,71,72,73,74,75], they are used solely as external contextual literature and are not treated as primary evidence for cyclic fatigue outcomes.

### 4.1. Mechanistic Interpretation of Cross-Sectional Geometry

The most important mechanistic implication of cross-sectional geometry is its effect on bending stiffness and stress distribution during repeated rotation or reciprocation in curved canals. During cyclic fatigue testing, the instrument segment located at the maximum canal curvature is exposed to alternating tensile and compressive stresses. A design with a smaller core and lower metal mass may bend more easily and generate lower restoring forces against the canal wall. This can reduce the amplitude of repeated bending stresses and delay crack initiation and propagation. In contrast, larger and more massive cores increase stiffness and may improve torsional resistance, but they may also increase bending stresses in curved canals [32,44,55,66].

This interpretation resolves the apparent contradiction between smaller cross-sectional area and higher fatigue resistance. A smaller section may experience higher nominal stress if the same external load is applied. However, cyclic fatigue tests of endodontic instruments are usually curvature-controlled rather than simple load-controlled tests. In curved canals, a more flexible instrument does not necessarily experience the same bending moment as a stiffer instrument, because it adapts more easily to the curvature and may reduce canal-wall contact forces. Therefore, the favorable behavior of low-mass S-shaped or double-S-shaped designs should be understood primarily as a consequence of reduced bending stiffness and optimized metal distribution, not as a direct increase in intrinsic material fatigue strength.

### 4.2. Influence of S-Shaped and Double-S-Shaped Cross-Sections on Fatigue Resistance

S-shaped and double-S-shaped cross-sections were most frequently associated with favorable cyclic fatigue performance, particularly in studies that directly assessed cross-sectional geometry or area [31,32,35,38,41,43,44,45,47]. These designs commonly reduce core mass and increase flexibility, which may improve their ability to withstand repeated bending. However, some triangular, rectangular, parallelogram, square, or variable designs also performed favorably when combined with advantageous heat treatment, reduced taper, controlled-memory alloys, or optimized core design [33,36,37,39,50,51]. S-shaped geometry should therefore be regarded as a favorable design pattern rather than an independent guarantee of superior fatigue resistance.

### 4.3. Materials-Science Perspective: Alloy Phase Composition and Manufacturing Technology

From a materials-science perspective, geometry must be interpreted together with NiTi microstructure and processing. Conventional austenitic instruments are generally stiffer, whereas instruments containing a higher proportion of martensite or R-phase after thermomechanical treatment usually show lower elastic modulus and greater flexibility. In the included evidence, heat treatment and manufacturing method materially modified fatigue behavior even when geometric design was explicitly evaluated [33,36,37,39,50,51,52].

Manufacturing quality and surface condition are also relevant because cyclic fatigue failure often begins at surface irregularities, machining grooves, microcracks, or cutting-edge defects. EDM manufacturing and other surface-modifying processes may influence crack initiation by changing surface topography, residual stresses, and local microstructure. Therefore, fatigue resistance is the result of an interaction between geometry-driven stress distribution and material-driven crack initiation and propagation. This is why direct comparisons between commercial systems should be interpreted cautiously unless alloy, heat treatment, size, taper, and kinematics are controlled.

### 4.4. Trade-Off Between Cyclic Fatigue and Torsional Resistance

The reviewed evidence confirms a clinically important trade-off between cyclic fatigue resistance and torsional resistance. Instruments with reduced core mass are usually more flexible and may perform better in curved canals, but this can be accompanied by lower torsional strength. Conversely, instruments with larger cross-sectional area and larger core diameter may better tolerate torsional stress when the instrument binds in a narrow canal, but they may be less resistant to repeated bending fatigue [32,44,45,55,66]. Consequently, the optimal cross-sectional design depends on the intended mechanical balance. Highly flexible, low-core instruments may be safer in severely curved canals, while larger-core instruments may offer greater torsional safety in canals with high frictional engagement.

### 4.5. Comparability of TTF and NCF Outcomes

The included studies frequently reported cyclic fatigue resistance as TTF, NCF, or both. These outcomes are related, but they are not always directly comparable because NCF depends on the rotational speed or reciprocating cycle frequency used during testing. In addition, temperature, canal curvature angle, radius of curvature, static versus dynamic testing, artificial canal material, lubrication, and instrument motion all influence fatigue life. For this reason, this review interpreted TTF and NCF primarily within the context of individual study designs rather than as universally comparable quantitative endpoints. These methodological differences also limited the feasibility of meta-analysis.

### 4.6. Flat-Side and Laterally Flattened Designs

Flat-side and laterally flattened designs did not show a consistent advantage in cyclic fatigue resistance [30,31,52]. Although lateral flattening may reduce the contact area between the file and canal wall, it may also modify metal distribution, cutting-edge geometry, and local stress concentration. In the studies included in this review, flat-side or laterally flattened instruments did not consistently outperform conventional or S-shaped instruments and, in some comparisons, demonstrated lower fatigue resistance. Therefore, lateral flattening alone should not be considered a reliable strategy to improve cyclic fatigue behavior.

### 4.7. Limitations and Future Research Directions

This review has several limitations. First, all included studies were in vitro investigations; therefore, their findings cannot be directly extrapolated to clinical conditions. Second, although generic commercial-system comparisons were excluded, several eligible geometry-focused studies still varied in more than one factor, including alloy composition, thermomechanical treatment, taper, dimensions, manufacturing process, surface condition, pitch, or kinematics. Consequently, the independent effect of cross-sectional geometry remains uncertain in part of the evidence base. Third, cyclic fatigue protocols varied considerably in terms of canal curvature angle and radius, testing temperature, motion kinematics, and outcome reporting. Fourth, most studies did not provide sufficiently comparable quantitative data to permit a meta-analysis. In addition, detailed geometric descriptors, such as core diameter, corner radius, flute depth, and cutting-edge angular orientation, were inconsistently reported and were rarely quantified using standardized methods. Therefore, no cross-study correlation between these parameters and TTF or NCF could be performed. Finally, reporting bias and certainty of evidence were not formally assessed because of the small and heterogeneous body of laboratory evidence.

Future studies intended to determine the independent influence of cross-sectional geometry should use purpose-designed, geometry-isolating in vitro comparisons in which instruments differ only in the prespecified geometric feature. Tip size, taper, longitudinal profile, pitch, alloy composition, thermomechanical treatment, manufacturing process, surface condition, and motion kinematics should be identical or experimentally controlled. Geometry should be quantified using micro-computed tomography or comparable three-dimensional morphometric methods, including measurements of cross-sectional area, core diameter and volume, metal mass, flute depth, corner radius and configuration, and cutting-edge angular orientation at predefined levels along the active portion of the instrument. Metallurgical equivalence and surface condition should also be verified using appropriate analytical methods. Cyclic fatigue testing should be performed under identical, prespecified, and clinically relevant conditions, including testing temperature, canal curvature angle and radius, canal material, lubrication, axial motion, and rotational or reciprocating parameters. The protocol should include an a priori sample-size calculation, random allocation, blinded or automated outcome assessment where feasible, balanced sampling from more than one production batch, and standardized reporting of TTF, NCF, fragment length, effect sizes, and measures of variability. These geometric parameters should then be correlated directly with fatigue outcomes under standardized testing conditions. Factorial designs may subsequently be used to investigate interactions between geometry and material, dimensional, or operating variables, whereas finite element analysis should be regarded as a complementary mechanistic method rather than a substitute for experimental cyclic fatigue testing. Finally, well-designed retrospective and prospective clinical studies, as well as analyses of incident-report databases, are needed to determine whether laboratory differences in fatigue resistance translate into clinically meaningful reductions in instrument separation during endodontic treatment.

### 4.8. Practical Interpretation

From a practical perspective, no single cross-sectional design can be considered universally superior. The available evidence supports a multifactorial interpretation: S-shaped or double-S-shaped designs are often favorable for cyclic fatigue resistance because they reduce core mass and bending stiffness, but their clinical usefulness depends on alloy treatment, taper, manufacturing quality, and motion kinematics. Instrument selection should therefore consider canal curvature, risk of torsional binding, desired flexibility, and the combined geometric and metallurgical design of the file system.

## 5. Conclusions

Cross-sectional geometry is associated with the cyclic fatigue resistance of NiTi endodontic instruments, but the evidence does not support interpreting it independently of material, dimensional, manufacturing, and operating characteristics. Across the 23 eligible studies, reduced metal mass, smaller core volume, lower cross-sectional area, and greater flexibility were most consistently associated with higher cyclic fatigue resistance in curved canals. These characteristics were frequently present in S-shaped and double-S-shaped designs. The association is mechanistically compatible with reduced bending stiffness, improved adaptation to canal curvature, and lower canal-wall contact forces; it does not prove increased intrinsic material fatigue strength. No cross-sectional design was universally superior. Triangular, rectangular, parallelogram, square, or variable designs also performed favorably when combined with advantageous alloy phase composition, thermomechanical treatment, optimized taper, or advanced manufacturing. Flat-side designs did not show a consistent advantage. Cyclic fatigue resistance should therefore be regarded as a multifactorial property. Future studies should use standardized protocols and controlled comparisons that isolate geometry from metallurgical and dimensional confounders.

## Figures and Tables

**Table 1:** Interpretative framework for factors influencing cyclic fatigue resistance of NiTi endodontic instruments.

Factor Level	Key Variables	Mechanistic Role	Expected Effect on Cyclic Fatigue	Interpretation in This Review	References
Cross-sectional geometry	Shape, core mass, cross-sectional area, off-center design, cutting edges	Controls bending stiffness, canal-wall contact, and stress concentration	Lower core mass often improves fatigue resistance in curved canals	Geometry is important but not independent	[32,35,38,41,43,47]
Metallurgy and phase composition	Austenite, martensite, R-phase, transformation temperatures	Determines elastic modulus, flexibility, and ability to accommodate repeated bending	Martensitic/R-phase or controlled-memory instruments often show higher resistance	May strengthen or outweigh geometric effects	[33,36,37,50,51]
Thermomechanical treatment and manufacturing	M-Wire, Blue, Gold, CM wire, EDM, machining quality, surface defects	Affects crack initiation sites and microstructural stability	Smoother or EDM/heat-treated systems may resist fatigue better	Essential for Materials-oriented interpretation	[34,36,37,39,50,51,52]
Dimensions and taper	Tip size, taper, core diameter, active blade length	Controls stiffness and torsional safety	Larger core/taper may reduce fatigue resistance but improve torsional resistance	Explains contradictions between studies	[32,36,44]
Kinematics and testing conditions	Rotary vs. reciprocating motion, speed, temperature, canal curvature, radius	Controls number and amplitude of stress cycles	Reciprocation and less severe curvature often increase fatigue life	Limits direct comparison of TTF and NCF between studies	[31,33,41,44,52]
Clinical trade-off	Flexibility, cutting efficiency, torsional resistance, debris removal	Determines balance between safety in curved canals and resistance to binding	No single cross-section is optimal for all conditions	Instrument choice should be multifactorial	[32,44,50,52]

**Table 2:** QUIN quality assessment of the 23 studies included in the qualitative synthesis. Studies are arranged alphabetically by the first author’s surname.

Authors	1	2	3	4	5	6	7	8	9	10	11	12	Score
Alcalde et al. [44] 2018	Yes	Yes	Yes	Yes	Yes	No	No	Yes	No	No	Yes	Yes	66.67
Ersoy et al. [39] 2016	Yes	No	Yes	Yes	Yes	No	No	Yes	No	No	Yes	Yes	58.33
Aranguren et al. [51] 2026	Yes	Yes	Yes	Yes	Yes	No	No	Yes	No	No	Yes	Yes	66.67
Brisighello et al. [40] 2019	Yes	No	Yes	Yes	Yes	Yes	No	Yes	No	No	Yes	Yes	66.67
Bürklein et al. [36] 2021	Yes	Yes	Yes	Yes	Yes	No	No	Yes	No	No	Yes	Yes	66.67
Di Nardo et al. [38] 2020	Yes	No	Yes	Yes	Yes	No	No	Yes	No	No	Yes	Yes	66.67
Faus-Llácer et al. [35] 2021	Yes	No	Yes	Yes	Yes	No	No	Yes	No	No	Yes	Yes	58.33
Grande et al. [47] 2006	Yes	No	Yes	Yes	Yes	No	No	Yes	No	No	Yes	Yes	58.33
Jeong et al. [30] 2024	Yes	Yes	Yes	Yes	Yes	No	No	Yes	No	No	Yes	Yes	66.67
Jusku et al. [34] 2021	Yes	No	Yes	Yes	Yes	No	No	Yes	No	No	Yes	Yes	58.33
Kaval et al. [45] 2017	Yes	No	Yes	Yes	Yes	No	No	Yes	No	No	Yes	Yes	58.33
Martins et al. [43] 2022	Yes	Yes	Yes	Yes	Yes	No	No	Yes	No	No	Yes	Yes	66.67
Martins et al. [52] 2023	Yes	Yes	Yes	Yes	Yes	No	No	Yes	No	No	Yes	Yes	66.67
Oh et al. [46] 2010	Yes	No	Yes	Yes	Yes	No	No	Yes	No	No	Yes	Yes	58.33
Reddy et al. [42] 2014	Yes	No	Yes	Yes	Yes	No	No	Yes	No	No	Yes	Yes	58.33
Sekar et al. [41] 2016	Yes	No	Yes	Yes	Yes	No	No	Yes	No	No	Yes	Yes	58.33
Silva et al. [37] 2020	Yes	Yes	Yes	Yes	Yes	No	No	Yes	No	No	Yes	Yes	66.67
Silva et al. [50] 2024	Yes	Yes	Yes	Yes	Yes	No	No	Yes	No	No	Yes	Yes	66.67
Tadano et al. [31] 2026	Yes	Yes	Yes	Yes	Yes	Yes	No	Yes	No	No	Yes	Yes	75.00
Teves-Cordova et al. [33] 2025	Yes	Yes	Yes	Yes	Yes	No	No	Yes	No	No	Yes	Yes	66.67
Tripi et al. [48] 2006	Yes	No	Yes	Yes	Yes	No	No	Yes	No	No	Yes	Yes	58.33
Vieira et al. [32] 2025	Yes	Yes	Yes	Yes	Yes	No	No	Yes	No	No	Yes	Yes	66.67
Yao et al. [49] 2006	Yes	Yes	Yes	Yes	Yes	No	No	Yes	No	No	Yes	Yes	66.67

1. Clearly stated aims/objectives; 2. Detailed explanation of sample size calculation; 3. Detailed explanation of sampling technique; 4. Details of comparison group; 5. Detailed explanation of methodology; 6. Operator details; 7. Randomization; 8. Method of measurement of outcome; 9. Outcome assessor details; 10. Blinding; 11. Statistical analysis; 12. Presentation of results.

## Data Availability

No new data were created or analyzed in this study. Data sharing is not applicable to this article.

## Supplementary Materials

The following supporting information can be downloaded at: https://www.mdpi.com/article/10.3390/jfb17080399/s1, Supplementary Table S1. PRISMA 2020 main and abstracts checklists; Table S2. Detailed study-level characteristics of the 23 studies included in the qualitative synthesis, organized alphabetically by first author and including key testing variables and quantitative cyclic fatigue outcomes.
