# Supplementary material for: Association Between Cross-Sectional Geometry and Cyclic Fatigue Resistance of Nickel–Titanium Endodontic Instruments: A Systematic Review
Source: J Funct Biomater. 2026 Aug 12;17(8):399. doi: 10.3390/jfb17080399 (PMC13514270; doi:10.3390/jfb17080399)
Supplement: Supplementary file 1 [file jfb-17-00399-s001.zip › jfb-4443096-supplementary.pdf]

Supplementary Table S1. PRISMA 2020 Main Checklist

Topic	No.	Item	Location where item is reported
**TITLE**			
**Title**	1	Identify the report as a systematic review.	1
**ABSTRACT**			
**Abstract**	2	See the PRISMA 2020 for Abstracts checklist	1
**INTRODUCTION**			
**Rationale**	3	Describe the rationale for the review in the context of existing knowledge.	1–3
**Objectives**	4	Provide an explicit statement of the objective(s) or question(s) the review addresses.	3
**METHODS**			
**Eligibility criteria**	5	Specify the inclusion and exclusion criteria for the review and how studies were grouped for the syntheses.	4–5
**Information sources**	6	Specify all databases, registers, websites, organisations, reference lists and other sources searched or consulted to identify studies. Specify the date when each source was last searched or consulted.	5
**Search strategy**	7	Present the full search strategies for all databases, registers and websites, including any filters and limits used.	5–6
**Selection process**	8	Specify the methods used to decide whether a study met the inclusion criteria of the review, including how many reviewers screened each record and each report retrieved, whether they worked independently, and if applicable, details of automation tools used in the process.	5–6
**Data collection process**	9	Specify the methods used to collect data from reports, including how many reviewers collected data from each report, whether they worked independently, any processes for obtaining or confirming data from study investigators, and if applicable, details of automation tools used in the process.	6

Topic	No.	Item	Location where item is reported
**Data items**	10a	List and define all outcomes for which data were sought. Specify whether all results that were compatible with each outcome domain in each study were sought (e.g. for all measures, time points, analyses), and if not, the methods used to decide which results to collect.	3, 6
	10b	List and define all other variables for which data were sought (e.g. participant and intervention characteristics, funding sources). Describe any assumptions made about any missing or unclear information.	6
**Study risk of bias assessment**	11	Specify the methods used to assess risk of bias in the included studies, including details of the tool(s) used, how many reviewers assessed each study and whether they worked independently, and if applicable, details of automation tools used in the process.	6
**Effect measures**	12	Specify for each outcome the effect measure(s) (e.g. risk ratio, mean difference) used in the synthesis or presentation of results.	3, 7
**Synthesis methods**	13a	Describe the processes used to decide which studies were eligible for each synthesis (e.g. tabulating the study intervention characteristics and comparing against the planned groups for each synthesis (item 5)).	3–7
	13b	Describe any methods required to prepare the data for presentation or synthesis, such as handling of missing summary statistics, or data conversions.	N/A
	13c	Describe any methods used to tabulate or visually display results of individual studies and syntheses.	4, 7–12; Tables 1–2; Figure 2; Supplementary Table S2
	13d	Describe any methods used to synthesize results and provide a rationale for the choice(s). If meta-analysis was performed, describe the model(s), method(s) to identify the presence and extent of statistical heterogeneity, and software package(s) used.	3–4, 7–10
	13e	Describe any methods used to explore possible causes of heterogeneity among study results (e.g. subgroup analysis, meta-regression).	N/A
	13f	Describe any sensitivity analyses conducted to assess robustness of the synthesized results.	N/A
**Reporting bias assessment**	14	Describe any methods used to assess risk of bias due to missing results in a synthesis (arising from reporting biases).	6
**Certainty assessment**	15	Describe any methods used to assess certainty (or confidence) in the body of evidence for an outcome.	6

Topic	No.	Item	Location where item is reported
**RESULTS**			
**Study selection**	16a	Describe the results of the search and selection process, from the number of records identified in the search to the number of studies included in the review, ideally using a flow diagram.	4, 7; Figure 2
	16b	Cite studies that might appear to meet the inclusion criteria, but which were excluded, and explain why they were excluded.	7, 13
**Study characteristics**	17	Cite each included study and present its characteristics.	7–10; Supplementary Table S2
**Risk of bias in studies**	18	Present assessments of risk of bias for each included study.	10–12; Table 2
**Results of individual studies**	19	For all outcomes, present, for each study: (a) summary statistics for each group (where appropriate) and (b) an effect estimate and its precision (e.g. confidence/credible interval), ideally using structured tables or plots.	Supplementary Table S2
**Results of syntheses**	20a	For each synthesis, briefly summarise the characteristics and risk of bias among contributing studies.	7–12; Tables 1–2; Supplementary Table S2
	20b	Present results of all statistical syntheses conducted. If meta-analysis was done, present for each the summary estimate and its precision (e.g. confidence/credible interval) and measures of statistical heterogeneity. If comparing groups, describe the direction of the effect.	N/A
	20c	Present results of all investigations of possible causes of heterogeneity among study results.	N/A
	20d	Present results of all sensitivity analyses conducted to assess the robustness of the synthesized results.	N/A
**Reporting biases**	21	Present assessments of risk of bias due to missing results (arising from reporting biases) for each synthesis assessed.	Not assessed; see p. 6
**Certainty of evidence**	22	Present assessments of certainty (or confidence) in the body of evidence for each outcome assessed.	Not assessed; see p. 6
**DISCUSSION**			
**Discussion**	23a	Provide a general interpretation of the results in the context of other evidence.	13–15

Topic	No.	Item	Location where item is reported
	23b	Discuss any limitations of the evidence included in the review.	15
	23c	Discuss any limitations of the review processes used.	15
	23d	Discuss implications of the results for practice, policy, and future research.	15
**OTHER INFORMATION**			
**Registration and protocol**	24a	Provide registration information for the review, including register name and registration number, or state that the review was not registered.	4
	24b	Indicate where the review protocol can be accessed, or state that a protocol was not prepared.	4
	24c	Describe and explain any amendments to information provided at registration or in the protocol.	6
**Support**	25	Describe sources of financial or non-financial support for the review, and the role of the funders or sponsors in the review.	16
**Competing interests**	26	Declare any competing interests of review authors.	16
**Availability of data, code and other materials**	27	Report which of the following are publicly available and where they can be found: template data collection forms; data extracted from included studies; data used for all analyses; analytic code; any other materials used in the review.	16

PRISMA 2020 for Abstracts Checklist

Topic	No.	Item	Reported?
**TITLE**			
**Title**	1	Identify the report as a systematic review.	Yes
**BACKGROUND**			
**Objectives**	2	Provide an explicit statement of the main objective(s) or question(s) the review addresses.	Yes
**METHODS**			
**Eligibility criteria**	3	Specify the inclusion and exclusion criteria for the review.	Yes
**Information sources**	4	Specify the information sources (e.g. databases, registers) used to identify studies and the date when each was last searched.	Yes
**Risk of bias**	5	Specify the methods used to assess risk of bias in the included studies.	Yes
**Synthesis of results**	6	Specify the methods used to present and synthesize results.	Yes
**RESULTS**			
**Included studies**	7	Give the total number of included studies and participants and summarise relevant characteristics of studies.	Yes
**Synthesis of results**	8	Present results for main outcomes, preferably indicating the number of included studies and participants for each. If meta-analysis was done, report the summary estimate and confidence/credible interval. If comparing groups, indicate the direction of the effect (i.e. which group is favoured).	Yes
**DISCUSSION**			
**Limitations of evidence**	9	Provide a brief summary of the limitations of the evidence included in the review (e.g. study risk of bias, inconsistency and imprecision).	Yes
**Interpretation**	10	Provide a general interpretation of the results and important implications.	Yes

Topic	No.	Item	Reported?
**OTHER**			
**Funding**	11	Specify the primary source of funding for the review.	Yes
**Registration**	12	Provide the register name and registration number.	Yes

From: Page, M.J.; McKenzie, J.E.; Bossuyt, P.M.; Boutron, I.; Hoffmann, T.C.; Mulrow, C.D.; Shamseer, L.; Tetzlaff, J.M.; Akl, E.A.; Brennan, S.E.; et al. The PRISMA 2020 Statement: An Updated Guideline for Reporting Systematic Reviews. BMJ 2021, 372, n71. https://doi.org/10.1136/bmj.n71[26] . For more information, visit: www.prisma-statement.org

Supplementary Table S2. Detailed study-level characteristics of the 23 studies included in the qualitative synthesis.

Study	Study focus	Comparison and assessment	Key fatigue-related findings
Alcalde et al. [44] 2018	To investigate cyclic and torsional fatigue resistance among three reciprocating instruments: Reciproc Blue Prodesign R and WaveOne Gold.	Reciprocating instruments tested: Reciproc Blue, ProDesign R, WaveOne Gold.; Cyclic fatigue and torsional resistance tests. SEM analysis of fractured surfaces. Cross-sectional design evaluated at different distances from the tip	Cyclic fatigue resistance: ProDesign R > Reciproc Blue > WaveOne Gold; Torsional strength: Reciproc Blue ≈ WaveOne Gold > ProDesign R; Flexibility: ProDesign R ↑ > Reciproc Blue > WaveOne Gold; Fragment length: no significant differences between groups.; Cross-sectional area (5 mm from tip): ProDesign R ↓ WaveOne Gold ↑; SEM: typical ductile cyclic fatigue features and characteristic torsional fracture damage.
Ersoy et al. [39] 2016	To examine two endodontic files with different cross-sectional shapes HyFlex CM and HyFlex EDM using finite element analysis (FEA) and their response to bending fatigue and torsional stress.	NiTi files tested: HyFlex EDM vs HyFlex CM (40/.04).; Cyclic fatigue test until fracture.; Torsional resistance test under rotational stress until failure.; Infrared thermography used to record temperature changes during crack formation/fracture	Cyclic fatigue resistance: HyFlex EDM) vs HyFlex CM ↓; Torsional resistance: HyFlex EDM ↑ time to fracture and ↑ angle of rotation before failure.; Stress concentration: highest near the instrument tip in both groups.; Fracture phase: failure occurred in the martensitic phase.
Aranguren et al. [51]2026	Assessent of the mechanical efficiency of NiTi rotary fil es with equivalent tip diameters but diverse compositions and morphology.	Tested instruments: WaveOne I and WaveOne II (WaveOne Gold) with different heat-treated NiTi alloys compared. Torsional resistance: in artificial canal and rotated counterclockwise until fracture.; Bending test; SEM	Fatigue resistance: Gold-treated instruments ↑; SE-Wire ↓; Flexibility: WO II/Gold ↑ SE-Wire ↓; Torsional strength: WO I ↑; Angular deformation: WO II ↑; Bending resistance: WO II ↓ SE-Wire ↑; SEM: SE-Wire showed more ductile fracture features; Gold (especially WO II) showed more brittle fracture behavior.

Study	Study focus	Comparison and assessment	Key fatigue-related findings
Brisighello et al. [40] 2019	To investigate whether the cross-section design and the number of uses influence the cyclic fatigue resistance of rotary files.	Tested instruments: K3 Endo and Endo Sequence.; Groups based on use frequency: 1×, 3×, or 5×. Canal preparation in simulated curved canals Dynamic cyclic fatigue test with pecking motion until fracture. Measured: time to failure	The number of times the files were used did not significantly affect their cyclic fatigue resistance, Cyclic fatigue resistance: K3 Endo ↑ vs Endo Sequence.
Bürklein et al. [36] 2021	To evaluate how core mass and alloy type influence the cyclic fatigue resistance of endodontic NiTi instruments that share the same outer diameter but differ in cross-sectional design.	120 NiTi rotary instruments: F360, Twisted File, JIZAI, TruNatomy, Mtwo, HyFlex OneFile.; 3D-printed artificial canals,; dynamic cyclic fatigue test using torque-controlled motor; Measured: time to fracture, NCF, fragment length.; Laser scanning microscopy: fracture mode analysis and exclusion of torsional failure	Cyclic fatigue (0.04 taper):; JIZAI 0.04 > F360 > Twisted File > TruNatomy; Cyclic fatigue (≥0.06 taper): HyFlex OneFile + JIZAI 0.06 ↑ resistance vs Mtwo;; Fragment length: HyFlex OneFile ↑ the longest (≥0.06 taper); TruNatomy ↓ the shortest (0.04 taper).; Fracture behavior: influenced by cross-sectional design and core diameter. Fractures consistent with cyclic fatigue failure.
Di Nardo et al. [38] 2020	To investigate the influence of the cross-sectional design of the instrument on the cyclic fatigue resistance.	Tested instruments: S-One vs AF Blue S4 (25/.06).; Cyclic fatigue test in stainless-steel artificial canal	Cyclic fatigue resistance: S-One ↑ (longer time to fracture) vs AF Blue S4 ↓; Fragment length: no significant differences between groups.; SEM: typical cyclic fatigue fracture features; failure due to repeated bending stresses.
Faus-Llácer et al. [35] 2021	To examine the influence of cross-sectional design on the dynamic cyclic fatigue resistance of NiTi endodontic rotary files.	Tested instruments: Mtwo, T Pro E1, E2, E4.; SEM: surface morphology; EDX: elemental composition.; Dynamic cyclic fatigue test in stainless-steel artificial canal with axial pecking motion.; Time to fracture and NCF recorded	No structural defects detected except minor debris in T Pro E2.; SEM: differences in cross-sectional design and pitch.; EDX: alloy composition differences; T Pro files contained aluminum.; Cyclic fatigue resistance: Mtwo> T Pro E1 > T Pro E2 > T Pro E4
Grande et al. [47] 2006	Evaluation of the correlation between the design of two NiTi rotary systems (Mtwo and ProTaper) and their fatigue durability under cyclic fatigue loading in artificial root canals.	Instruments tested: ProTaper (S1, S2, F1, F2, F3) and Mtwo (10/.04–40/.04/.05/.06).; Micro-CT: 3D volumetric analysis of instrument geometry.; Cyclic fatigue and SEM of fractured fragments	Cyclic fatigue resistance: ↓ with increasing instrument size and metal volume.; Fatigue resistance: Mtwo ↑; Curvature radius: smaller radius ↓ fatigue life; larger radius ↑ fatigue resistance.; SEM: similar characteristic cyclic fatigue fracture features in all instruments.
Jeong et al. [30] 2024	To compare effects of a modified nickel–titanium (NiTi) rotary instrument design with laterally flattened flutes.	NiTi rotary with lateral flattened flutes (rotary) and conventional without flattening; Cyclic Fatigue was tested in artificial canal . Torsional test performed by maximum torque, angular deflection and toughness. The fracture surfaces were examined by scanning electron microscopy (SEM)	Cyclic fatigue resistance: conventional instruments > rotary instruments; No significant differences in fragment length, maximum torque, or angular deflection.; SEM: both groups showed typical cyclic/torsional fracture features; cracks initiated at cutting edges.
Jusku et al. [34] 2021	To investigate how cross-sectional area and surface roughness, along with other mechanical properties.	4 Reciprocating NiTi instruments tested: Unicone, Unicone Plus, Reciproc Blue, WaveOne Gold; Cyclic fatigue tested in curved stainless-steel canal measuring time to fracture.; Nanoindentation: hardness, elastic modulus, creep. Confocal microscopy for surface characterization	Cyclic fatigue resistance: Reciproc Blue and Unicone Plus ↑; Unicone ↓; surface roughness: Unicone Plus: ↓ (smoothest), WaveOne Gold: ↑; Unicone: ↑ hardness, ↓ creep.

Study	Study focus	Comparison and assessment	Key fatigue-related findings
Kaval et al. [45] 2017	Assessment of cyclic fatigue resistance: F6 SkyTaper, KSXF, new generation OneShape and TRUShape 3D conforming files.	10 instruments in each group tested: F6 SkyTaper, K3XF, OneShape, TRUShape 3D.; Cyclic fatigue test in stainless-steel artificial canal.; Files rotated with a low-torque motor at room temperature.; Cyclic fatigue resistance measured by time and NCF to fracture	Cyclic fatigue resistance: F6 SkyTaper > K3XF > TRUShape 3D > OneShape; Statistically significant differences observed among all groups.
Martins et al. [43] 2022	To evaluate which of the examined endodontic instruments (ProGlider, Edge Glide Path and R-Pilot) is more resistant to cyclic fatigue.	Instruments tested: ProGlider, Edge Glide Path, R-Pilot.; SEM: design and cross-sectional geometry analysis.; EDS: Ni/Ti composition.; DSC: phase transformation temperatures. Cyclic fatigue test in custom-made artificial canals	Blade design: ProGlider ↑ most blades; R-Pilot ↑ largest helical angle.; Cross-sections: ProGlider – square; Edge Glide Path – triangular; R-Pilot – S-shaped.; Surface quality: Edge Glide Path ↑ smoothest; Cyclic fatigue resistance: R-Pilot ↑ highest endurance; Edge Glide Path ↓ lowest.; Fragment length: no significant differences between groups.
Martins et al. [52] 2023	To compare flat-side heat-treated rotary single-file systems (AF F One Blue and Platinum V.EU) with reciprocating single-file systems (One Files Blue.	290 NiTi instruments tested rotary – AF F One Blue, Platinum V.EU; reciprocating – One Files Blue, Reciproc Blue, Reciproc.; Design analysis by stereomicroscopy and SEM: blade number, helical angle, cross-section, tip design, surface finishing/defects.; Metallurgy: EDS (Ni/Ti ratio) + DSC (phase transformation temperatures).; Cyclic fatigue; Torsional resistance: maximum torque and angle of rotation; flexibility: bending test; buckling resistance and microhardness: Vickers testing	Design: similar blade number and helical angle in all systems.; Flat-side instruments: manufacturing irregularities, surface defects, incomplete S-shaped cross-section.; Reciprocating instruments: smoother and more uniform surfaces.; Metallurgy: near-equiatomic NiTi; different phase transformation temperatures among systems.; Cyclic fatigue resistance: reciprocating systems ↑; Torsional resistance: reciprocating systems - ↑ torque and ↑ angle of rotation; Flexibility: Reciproc Blue ↑; Buckling resistance: Reciproc ↑; flat-side systems ↓; Microhardness: Reciproc ↑, AF F One Blue ↓
Oh et al. [46] 2010	Evaluation of impact manufacturing methodology and the cross-sectional area of NiTi rotary files on their cyclic fatigue resilience.	NiTi instruments tested: ProFile, RaCe, K3, TF.; Cyclic fatigue test in stainless-steel artificial canal.; Measured: time to fracture, NCF, fragment length; SEM	Cyclic fatigue resistance: TF > ProFile > RaCe ≈ K3.; ProFile higher fatigue resistance than K3.; Fragment length: similar in all groups.; Cross-sectional area: TF ↓ K3 ↑ (≈2× TF).; SEM: ProFile and K3 showed manufacturing defects, RaCe showed free of external defects and TF exhibited wrinkle-like parallel grooves.
Reddy et al. [42] 2014	Assessment and comparison the number of cycles to failure for 3 NiTi rotary files in simulated curved canals.	NiTi instruments tested: ProFile, K3 Endo, RaCe .; Cyclic fatigue testing in artificial canals with universal testing machine, endodontic motor and handpiece used. SEM (200×, 800×) for fracture mechanism analysis	Cyclic fatigue resistance (NCF): ProFile ↑ at both 5 mm and 10 mm curvature radii.; RaCe ↓ NCF; SEM: ProFile showed ↓ microvoids, dimples and smooth fracture edges, indicating slower fatigue progression.
Sekar et al. [41] 2016	Evaluation of cyclic fatigue resistance cross-sectional design on the cyclic fatigue resistance of NiTi rotary endodontic instruments in continuous rotation and reciprocating motion.	Tested instruments: Mtwo, One Shape, Revo-S SU under continuous rotation vs reciprocating motion. Cyclic fatigue until fracture.; SEM analysis of selected fractured samples. Longitudinal design assessed by thread count	Motion: reciprocating ↑ cyclic fatigue resistance vs continuous rotation.; Cyclic fatigue resistance: Mtwo > One Shape > Revo-S SU; SEM: ductile fracture mode.; Thread count: One Shape 14 > Revo-S SU 11 > Mtwo 7.

Study	Study focus	Comparison and assessment	Key fatigue-related findings
Silva et al. [37] 2020	To evaluate NeoNiti, HyFlex EDM, ProTaper Gold, and ProTaper Universal rotary systems in terms of cyclic fatigue resistance, torsional strength.	Files tested: NeoNiti A1, HyFlex EDM One File, ProTaper Gold F2, ProTaper Universal F2.; Cyclic fatigue: curved stainless-steel canal measured by TTF & NCF.; Torsional test: maximum torque and angle of rotation.; SEM/EDS: design, fracture surfaces, Ni/Ti composition.; DSC: austenite/martensite transformation temperatures.; Micro-CT shaping analysis on maxillary molars: unprepared surface area and shaping performance	Cyclic fatigue resistance: HyFlex EDM and NeoNiti A1 ↑ highest; ProTaper Universal F2 ↓.; Angular rotation to fracture: HyFlex EDM.; Torque to fracture: ProTaper Universal F2 ↑.; Metallurgy: NeoNiti/HyFlex EDM high-quality surfaces, different cross-sections; ProTaper Gold ↑ transformation temperatures.; EDS: equiatomic Ni-Ti composition.; SEM: typical ductile cyclic fatigue and torsional fracture patterns.
Silva et al. [50] 2024	To compare a novel multicoloured NiTi rotary system with four established heat-treated instruments. The evaluation focused on their geometric design.	300 NiTi rotary instruments in 5 systems: RCS Rainbow, Rotate, RaCe EVO, OneCurve, ProTaper Ultimate were analyzed.; Design: SEM, stereomicroscopy, 3D surface scanning (cross-section, blade morphology).; EDS and DSC metallurgical analysis.; Mechanical tests: cyclic fatigue, torsional (torque/angular deflection), bending, buckling, cutting efficiency.; Micro-CT shaping analysis before/after preparation: volume, surface area, untouched walls	RCS Rainbow: ↑ bending and buckling resistance,, ↓ torque resistance, ↓ flexibility, ↓ angular deflection.; OneCurve: ↑ cyclic fatigue resistance ↑ cutting efficiency; ↑ balanced performance.; RaCe EVO: ↑ angular rotation, ↓ fatigue resistance ↓ cutting ability.; ProTaper Ultimate: ↑ torsional resistance, ↓ fatigue resistance.; No significant differences in shaping ability and preparation outcomes.
Tadano et al. [31] 2026	To evaluate the influence of different cross-sectional designs of NiTi rotary instruments on smear layer removal from dentinal walls.	80 NiTi rotary files (Spin, S2, Rotate and Platinum V.EU) from 40 mandibular incisors sectioned and analyzed by SEM. Quantitative assessment using percentage of clean dentin surface).; Cyclic Fatigue was evaluated in stainless-steel canal. Dynamic rotational testing according to manufacturer settings	Smear layer: no intergroup difference;; Rotate system showed the lowest cyclic fatigue resistance and ↓ torque resistance, ↑ angular deflection.; Platinum V.EU Platinum V.EU ↑ fracture time, but lower NCF than Spin/S2.; SEM: ductile features with dimples. Torsional fractures: concentric abrasion marks and fibrous structures
Teves-Cordova et al. [33] 2025	Evaluation of the combined influence of metallurgical characteristics and cross-sectional design on the cyclic fatigue and torsional resistance.	240 NiTi rotary instruments (25/.06, 35/.04 from 6 systems evaluated:; Plex V (PV); ProFlexi File (PFF); MG3 Gold (MG3); RCS Rainbow One (RRO); AF FOne (AFR); Rotate; Cyclic fatigue in stainless-steel canal.; Torsional test: maximum torque + angular deflection.; DSC/XRD: phase transformation and austenite/martensite/R-phase analysis.; SEM: fractured surface	Cyclic fatigue resistance (NCF): RRO ↑; MG3/VDW ↓ in size 25; Size 35: PFF + RRO ↑ fatigue resistance.; Torque resistance: Plex V ↑ VDW/RRO ↓; Angular deflection: VDW ↑ (size 35); DSC/XRD: different phase transformation temperatures and austenite/martensite/R-phase proportions.; ↑ Martensite/R-phase → ↑ flexibility and fatigue resistance.; SEM: ductile microvoids (cyclic fatigue) and concentric abrasion /fibrous dimples (torsional shear failure).
Tripi et al. [48] 2006	Assessment of the influence of the design and surface treatment of Niti rotary instruments on the occurrence of flexural fractures.	Tested instruments: ProFile, RaCe (polished and unpolished), K3, Hero, Mtwo; Cyclic fatigue test in artificial curved canal measured by TTF; SEM: surface quality, defects, and fracture pattern analysis	Cyclic fatigue resistance: ProFile > RaCe and K3 > Hero, Mtwo, and unpolished RaCe; Electropolishing: ↑ fatigue resistance and ↓ surface defects and microcracks in RaCe instruments.; Instrument design and surface condition influenced fracture resistance.

Study	Study focus	Comparison and assessment	Key fatigue-related findings
Vieira et al. [32] 2025	In vitro evaluation of the isolated effect of cross-sectional geometry on the mechanical behaviour of NiTi rotary instruments.	2 rotary groups with identical size, taper, and heat treatment; the same size/taper, but different cross-section: S-shaped and triangular; Design analysis: blade length, spirals, helical angle.; DSC: phase transformation temperatures.; 3D geometry: metal mass volume + cross-sectional area.; Cyclic fatigue, torsional (torque/angular deflection; ISO 3630) and bending resistance evaluated.; SEM	Comparable metallurgical/thermal properties in both groups.; S-shaped: ↓ metal mass ↓ cross-sectional area, ↑ cyclic resistance, ↑ angular deflection, ↑ flexibility.; Triangular: ↑ maximum torque, ↑ bending resistance (stiffer).; SEM: ductile fracture (dimples)
Yao et al. [49] 2006	Assessment of the resistance of K3, ProFile and RaCe instruments to fracture during operation.	NiTi instruments tested: ProFile, K3, and RaCe with various sizes and tapers.; Cyclic fatigue measured: (TTF) and number of rotations/cycles to fracture.; SEM: surface morphology analysis	Cyclic fatigue resistance: K3 Endo ↑ (specially K3 25/.04) > ProFile > RaCe; File size, taper, and design influenced fracture resistance.; SEM: fractures mainly caused by cyclic fatigue
